# The Depth–Width Correlation for Shrinkage-Induced Cracks and Its Influence on Chloride Diffusion into Concrete

**DOI:** 10.3390/ma13122751

**Published:** 2020-06-17

**Authors:** Hongguang Zhu, Qingjie Huo, Jingchong Fan, Sen Pang, Hongyu Chen, Cheng Yi

**Affiliations:** School of Mechanics and Civil Engineering, China University of Mining and Technology, Beijing 100083, China; zhg@cumtb.edu.cn (H.Z.); sqt1800603056@student.cumtb.edu.cn (Q.H.); bqt1800603022@student.cumtb.edu.cn (J.F.); bqt1800603020@student.cumtb.edu.cn (H.C.); yc@cumtb.edu.cn (C.Y.)

**Keywords:** shrinkage-induced cracks, depth-width scaling, diffusion, chloride

## Abstract

This study examined the depth–width correlation of actual shrinkage-induced cracks and its influence on the diffusion properties of concrete. An experimental setup of restrained slabs was utilized to induce the shrinkage cracks, and the geometry characteristics were quantified with image analysis technology. The results indicated the depth–width scaling λ of shrinkage cracks increases with crack width and was almost constant when the crack width was approximately 0.3 mm or more, and the tip angle of shrinkage cracks is about 1–2 degrees. The diffusion coefficients of concretes were measured by a conductivity test method. A series-parallel composite model with λ was devised to evaluate the diffusivity of shrinking cracked concrete. It was shown that the equivalent diffusion coefficient depended greatly on the crack depth instead of the crack width, and it was found to be a nonlinear relationship versus the width combining with λ. The diffusion coefficient of the crack *D_cr_* was correlated to both crack width and λ, and increased with crack width. When the crack width is higher than 0.2 mm *D_cr_* becomes constant, where the value obtained was 87% of the diffusion coefficient in free solution.

## 1. Introduction

Steel corrosion induced by chloride is one of the major deterioration issues for reinforced concrete structures worldwide. Moreover, the chloride diffusion in cover concrete, a key point for the deterioration, is associated with increased importance to the design of concrete structures and studied by a great many investigators. Most studies are with sound (uncracked) concrete [1,2,3,4,5,6,7]. However, concrete structures are always serviced with cracks due to the weakness in tension. When the concrete undergoes dry ambient conditions, it shrinks, which induces internal tensile stresses and potential cracking, considering that reinforced concrete is usually strained. These cracks are the transport paths for aggressive ions penetrating into concrete. Therefore, it is significant to understand the realistic influence of shrinkage-induced cracks on chloride diffusion for the service-life prediction of concrete structures.

Different methods were employed to generate cracks in concrete in laboratory studies. Overall, the cracks could be distinguished into two groups: traversing cracks and non-traversing cracks. To induce cracks more realistically, the Brazilian or wedge splitting method is usually used to create traversing cracks, by which the cracks generated have a uniform length and depth [8]. Further, crack width is the only key parameter influencing chloride penetration. The approach to produce non-traversing cracks is a three- or four-point bending test [9] or shrinking concrete [10]. For this group, both the width and depth significantly influence the permeability of concrete [11], which varies along the crack length.

Because the shape can be relatively well-controlled by mechanical expansion, multiple works have been performed on the permeability properties of concrete with traversing cracks. In those researches, the traversing cracks are commonly simplified as parallel in hypothetical models [8,12], and the relationship between crack width and concrete permeability is concluded as proportional or quasi proportional correlation [8,13,14]. However, in engineering concrete, typical cracks, especially shrinkage cracks, are non-traversing. Hearn reported that the orientation and distribution of shrinkage cracks in concrete were effectively isotropic and have a significant influence on the coefficient of water permeability of specimens, whereas load-induced cracks were strongly oriented and localized but did not have a measurable effect on the permeability [15]. This indicates that the previous research conclusion of load cracks may not be suitable for shrinkage cracks. In addition, some studies show that the shrinkage cracks are developed randomly [10] and obey a certain distribution function [16]. However, it is difficult to ensure the validity of experimental data because the randomness leads to uncontrollable and unrepeatable crack shapes, and no meaningful regular conclusion has been reached. In terms of artificial non-traversing cracks, Marsavina et al. show some evidence that the higher mean chloride penetration depth is associated with higher crack depth and the influence of crack width on chloride penetration is less pronounced [11]. However, how to analyse and characterize the salient features of shrinkage cracks remain unclear and requires further study, especially regarding crack depths. 

Meanwhile, the “threshold crack width” [17,18] is introduced in many studies, below which the permeability of concrete is slightly affected [19]. Available data suggest that the threshold crack width varies significantly from 0.055 mm to 0.1 mm, and this range is substantially less than the limit of crack width allowable for concrete members in Chinese National Standards GB 50010 [20] or CEB-FIP Model Code 90 which is stipulated as 0.2 mm to prevent the corrosion of steel reinforcement. Therefore, it is necessary to discuss whether the allowable value of the crack width should be limited further.

This paper proposes to take a further step in understanding of the characterization of real shrinkage cracks, especially the crack depths and its effects on chloride ion diffusion into concrete. To simulate the dry-shrinking and induce various shrinkage cracks, an external restraining test method was developed in slabs. With image processing and analysis technology, the crack width, depth, and their correlation are all quantified statistics, and their influence on the chloride diffusion is studied by combining a theoretical model, analysis, and experimental methods. The availability of this research is indispensable to accurately evaluate the influence of shrinkage cracks on the concrete permeability.

## 2. Diffusion in Cracked Concrete—A Hypothetical Series—Parallel Model for Non-Traversing Cracks

When chloride diffusion is measured by a steady state migration test under electric field acceleration, the saturated concrete can be seen as an electrical conductor, and the method of a series-parallel connection of resistors in electricity can be used to study the diffusion process in cracked concrete. So, the total current passing through the concrete can be expressed as follows:

(1) $$I=JA=kD\frac{\Delta E}{H}A$$

where *D* is the diffusivity of chloride [m^2^/s], Δ*E* is the potential applied [V], *I* is the current [A], *H* is the concrete specimen (disc) thickness [m], *A* is the cross-section area of concrete specimen [m^2^], and *k* is a constant that determined by the test method.

**Figure 1:**
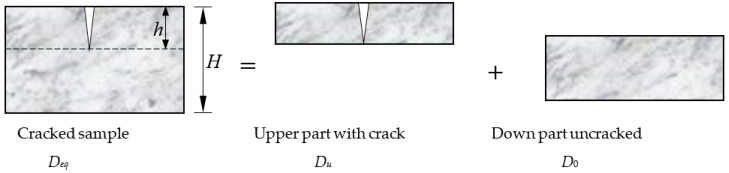
Partition hypothesis of resistance for cracked specimen.

Since the angle of the crack tip is extremely small, the crack is idealized as a straight channel. If the crack size length × width × depth = *l* × *w* × *h* (m^3^), the diameter of cylindrical cross section specimen is *b* (m). Then, the total potential drop of cracked concrete sample can be divided into two parts which are installed in series, as shown in Equation (2) and Figure 1:

(2) $$\Delta E_{t}=\Delta E_{u}+\Delta E_{d}.$$

Replacing Equation (1) in Equation (2), one finds Equation (3):

(3) $$k\frac{H}{D_{eq}}\frac{I}{A}=k\frac{h}{D_{u}}\frac{I_{u}}{A}+k\frac{\left(H-h\right)}{D_{0}}\frac{I_{d}}{A}.$$

For each part, the charge conservation equation leads to *I_u_* = *I_d_* = *I*, and Equation (3) may be rewritten as:

(4) $$\frac{D_{eq}}{D_{0}}=\frac{H}{h\times \frac{D_{0}}{D_{u}}+H-h}=\frac{1}{1+\left(\frac{D_{0}}{D_{u}}-1\right)\frac{h}{H}}$$

where *D_eq_*, Δ*E_t_*, *I* refer to the equivalent diffusion coefficient, the total potential drop, and the currents of cracked concrete (m^2^/s). *D_u_*, Δ*E_u_*, *I_u_* refer to the diffusion coefficient, the potential drop, and the currents of upper part concrete, respectively (m^2^/s). *D*_0_, Δ*E_d_*, and *I_d_* are the diffusion coefficient, the total potential drop, and the currents of down part uncracked concrete (m^2^/s), respectively.

**Figure 2:**
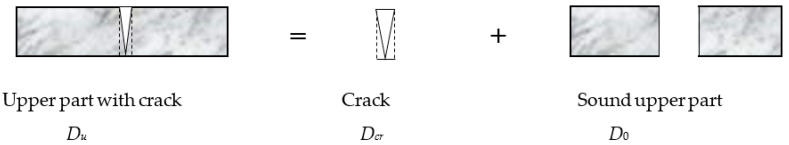
Partition hypothesis of resistance for the upper part with crack.

The resistance of upper part concrete with crack can be divided into two parts which are installed in parallel, as shown Figure 2.

Then, the total current through two parallel parts can be given as follows:

(5) $$I=I_{cr}+I_{s}$$

where *I_cr_* and *I_s_* are the currents through the crack and sound upper part concrete [A], respectively. Replacing Equation (1) in Equation (5), one finds Equation (6):

(6) $$\frac{D_{u}A}{kh}\Delta E_{u}=\frac{D_{0}A_{uncr}}{kh}\Delta E_{u}+\frac{D_{cr}A_{cr}}{kh}\Delta E_{u}$$

(7) $$\Rightarrow \frac{D_{u}}{D_{0}}=\frac{A_{uncr}}{A}+\frac{A_{cr}}{A}\frac{D_{cr}}{D_{0}}$$

where *D_cr_* is the diffusion coefficient through the crack (m^2^/s), while *A_cr_* and *A_uncr_* are the superficial areas of crack and uncracked upper part concrete (m^2^), respectively. Since *A_uncr_*/*A* ≈ 1, combining Equation (7) with Equation (4) and solving for *D_eq_*, yields

(8) $$\frac{D_{eq}}{D_{0}}=\frac{\frac{A_{cr}}{A}\frac{D_{cr}}{D_{0}}+1}{\frac{A_{cr}}{A}\frac{D_{cr}}{D_{0}}\left(1-\frac{h}{H}\right)+1}$$

For disc specimens, *A* = *πb*^2^/4. Equation (8) may be rewritten as

(9) $$\frac{D_{eq}}{D_{0}}=\frac{\frac{4}{\pi }\frac{w}{b}\frac{l}{b}\frac{D_{cr}}{D_{0}}+1}{\frac{4}{\pi }\frac{w}{b}\frac{l}{b}\frac{D_{cr}}{D_{0}}\left(1-\frac{h}{H}\right)+1}$$

Boundary condition check: uncracked state *h* = 0 or *w* = 0 or *l* = 0, *D_eq_* = *D*_0_, and traversing crack state *h* = *H*, *D_eq_* = *D_u_*. Equation (9) satisfies this.

Usually, the length of cracks in engineering is much larger than its width. To simplify the analysis, a section can be taken along the crack length for discussion. Equation (8) may be rewritten as

(10) $$\frac{D_{eq}}{D_{0}}=\frac{\frac{w}{b}\frac{D_{cr}}{D_{0}}+1}{\frac{w}{b}\frac{D_{cr}}{D_{0}}\left(1-\frac{h}{H}\right)+1}$$

**Figure 3:**
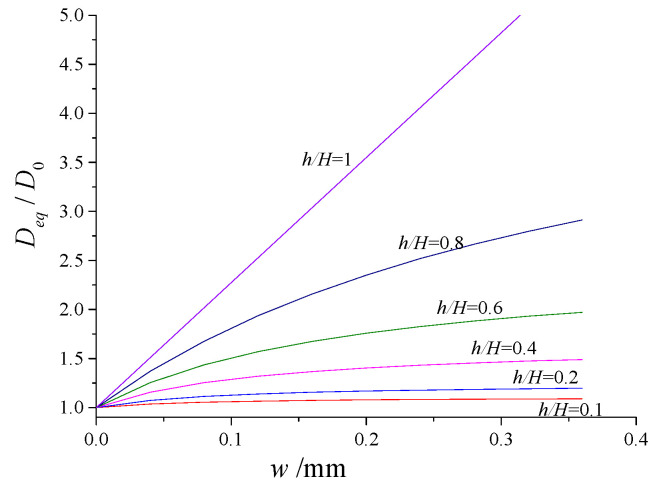
Variation of *D_eq_/D*_0_ as a function of *h/H* and *w* obtained from Equation (10).

According to previous research results, *D_cr_/D*_0_ is far greater than 1 for concrete (700–5000, Djerbi et al. [8]). Here, taking *D_cr_/D*_0_ = 1000 for example, the variation of equivalent diffusivity (*D_eq_/D*_0_) as a function of the crack relative depth *(h/H)* and width (*w*) is illustrated in Figure 3. 

As can be seen in this figure, when *h*/*H* < 1, e.g., the non-traversing cracks, the equivalent permeability coefficient has a nonlinear relationship with the crack width, and *D_eq_/D*_0_ increases with *w* but the growth rate is decreasing. When *h*/*H* = 1, e.g., the traversing cracks, the relationship evolves to be linear, which conforms to the results recently reported by Djerbi et al. [8].

The curves of Figure 3 also indicate that the effect of the crack tends to be more significant as the ratio *h/H* increases. This essentially means that the influence of crack depth will be relatively more important than crack width on the equivalent diffusivity for cracked concrete. It is therefore important to understand the depth characteristics of the shrinkage crack. Existing literature has revealed that, for opening-mode cracks, the depth and the width are related [21,22], such as shrinkage cracks in concrete. Moreover, crack depth is difficult to measure while the width is easy. That establishment of the relationship between width and depth to replace the depth measurement with width measurement can facilitate the engineering applications, so the scaling of depth to width is introduced. And we designed a series of experiments to obtain shrinkage cracks, measure the crack depth, width and their scaling and whose effects on chloride diffusion in concrete.

## 3. Experimental Program

### 3.1. Materials

The mix proportion used in the present study is listed in Table 1. For this mixture, the 42.5 ordinary Portland cement produced in Yanxin company (Herbei, China), and the local river sand as a fine aggregate with a fineness modulus of 2.70, was used. The cubic compressive strength of concrete was tested after 28 days of curing, and the average measured values were 44.3 MPa.

### 3.2. Preparation of Cracked Specimens

**Figure 4:**
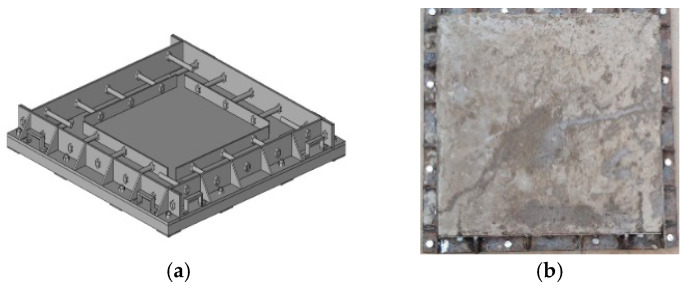
(**a**) The setup apparatus and (**b**) Casted concrete slab with external restraint.

A concrete mould of the external restrained slab was developed to generate shrinking cracks. The dimension of slabs is 500 × 500 × 50 mm. In order to simulate the shrinkage cracking of concrete slab in terms of practical engineering, using the maximum size of aggregate, we refer to the suggestions of Reference [23] and Chinese National Standards GB/T 50204 [24]: the maximum size of coarse aggregate may be 20–40 mm for solid concrete slab with thickness of 50–150 mm in concrete structure. And we select 20 mm as the maximum size. Drying was permitted on the top surfaces. The ribbed angle steel with the thickness of 12.5 mm was used at each edge of the slab to provide rigidity to the setup frame. In order to guarantee the horizontal restraint, four steel plates were respectively attached to those angle steels with coarse threaded bars before casting, making it possible for fresh concrete to harden inside. Figure 4 shows the setup and casted concrete in slab with the external restraint. 

Four slabs were casted. After casting, the slabs were drying for 3 days to induce cracks, exposed to a constant relative humidity of 25% ± 5% and at a constant room temperature of 35 °C inside a humidity-controlled chamber. Then, the slabs were cured with external restraint for 28 days in water, which could allow the concrete to hydrate further and release the uneven residual stress caused by drying shrinkage to prevent the crack from healing. The diamond wire saw cutting was used to reduce the extra damage to concrete, and samples of *ϕ* 100 mm cylindrical were obtained from the cracked parts of those slabs and cured for 3 months.

### 3.3. Chloride Diffusion Tests

**Figure 5:**
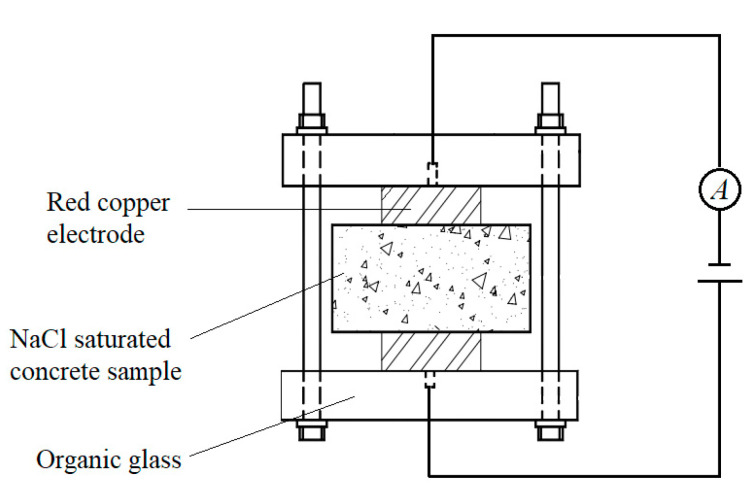
The schematic diagram of the test device of concrete conductivity method.

The concrete conductivity test method [1,25,26], shown in Figure 5, was adopted to evaluate the chloride diffusivity in concrete specimens, which can be finished within a few minutes. Prior to the migration test, the concrete specimens were cleaned and then vacuum saturated with a 4 mol/L NaCl solution. Afterwards, an external electrical potential of 1–10 V was applied across the specimen and the current was measured, before the chloride diffusion coefficient could be calculated by the Nernst-Einstein equation [27].

### 3.4. Crack Geometry Observations

**Figure 6:**
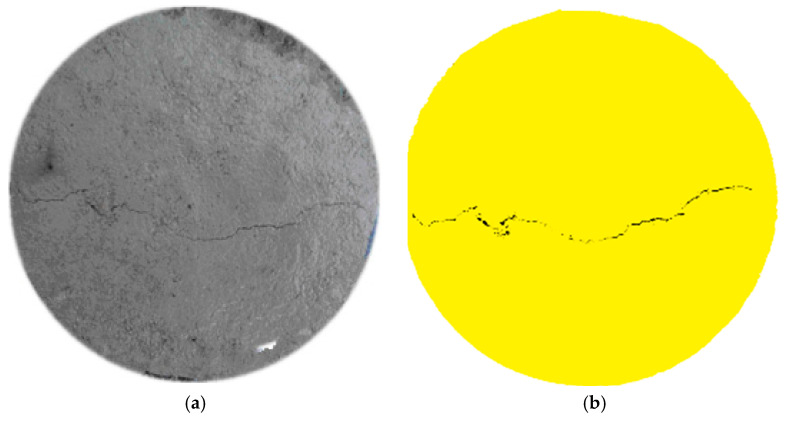
Distinguishing cracks using digital images. (**a**) Grey image of cracked specimens; (**b**) Crack distribution on the concrete.

The measured parameters were the crack length, the width and the depth. The crack length and width information were gathered by image analysis. First, we imaged the cracked specimens using a digital camera with 20 MP (megapixels). Then, we convert the RGB color image into a 256 gray level image and define the crack pixels as 1 and the background pixels as 0 to get a binary image. After that, we rebuilt the image with a smooth filter to suppress noise and remove small objects, and the geometrical shape of cracks in concrete was distinguished (Figure 6). Based on pixel analysis, the measurements of crack parameters were taken with a maximum width resolution of 20 μm.

**Figure 7:**
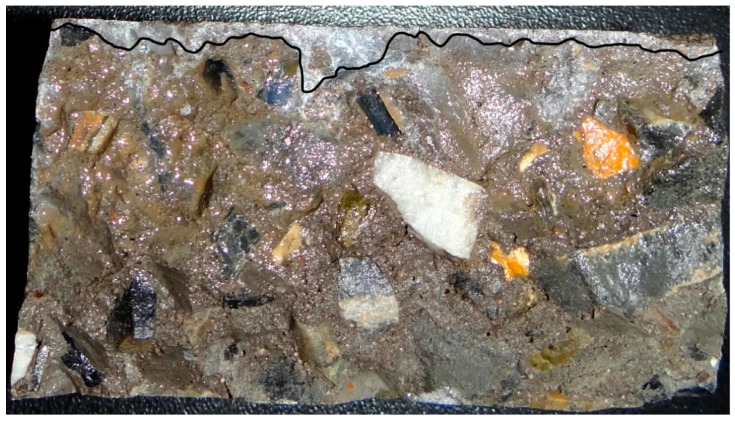
Measurement of crack depths.

The crack depth was measured after the diffusion test. A 0.1 mol/L AgNO_3_ solution was injected into the cracks to keep show of their outlines and then the specimens were split into two slices at the crack. In the end, the crack depth was measured at locations from the visible white silver chloride precipitation, as shown in Figure 7.

## 4. Correlation between the Crack Depth *h* and Width *w*

The scaling of depth to width is defined as follows:

(11) λ=h/w

First, we need to ensure the validity of the measured data before analysis, and this can be checked by a consistency test because of the randomness of shrinkage cracks. We use the chi-square method for checking. Through the experimental test, 64 sets of effective crack data were obtained, and which satisfies the general statistical requirements (>30). These data were statistically analyzed using SPSS software, and its statistical parameters are listed in Table 2.

**Figure 8:**
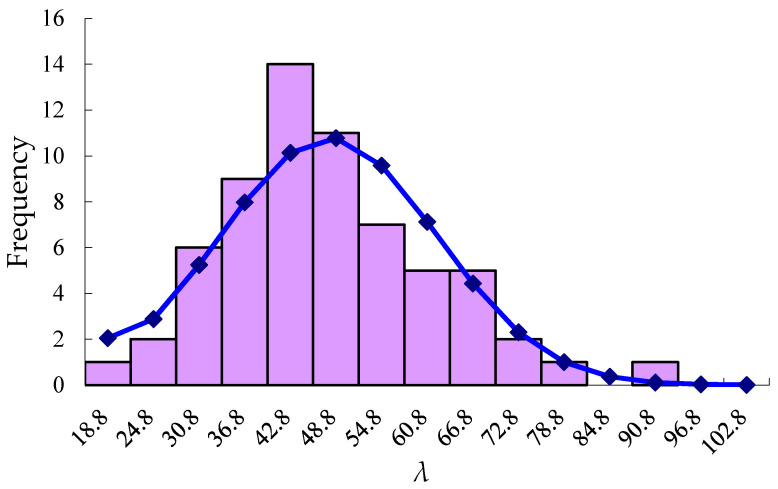
Statistics histogram of width-depth scaling of cracks.

The Chi-square statistical result is shown in Figure 8. When the confidence requirement is set as 95%, the theoretical threshold value χ2(10,0.95) = 18.31, and the statistical Chi-square value is 11.54 which is less than the theoretical threshold. Therefore, we accept the hypothesis that there is no significant difference for frequency distribution in different categories of λ, and this also indicates that the test method proposed in this paper can correctly simulate the generation of shrinkage cracks in concrete.

Then, a statistical analysis of collected λ data has been taken. The average value of λ is 44.90. If the section of shrinkage crack is triangular, the average crack tip angle can be obtained about 2.6 degrees. In Figure 8, total frequency of the seven bars 30.8–66.8 is 57, which accounts for 90% nearly. That is to say, the angle of shrinkage crack converted is mainly in the range of 1–2 degrees.

**Figure 9:**
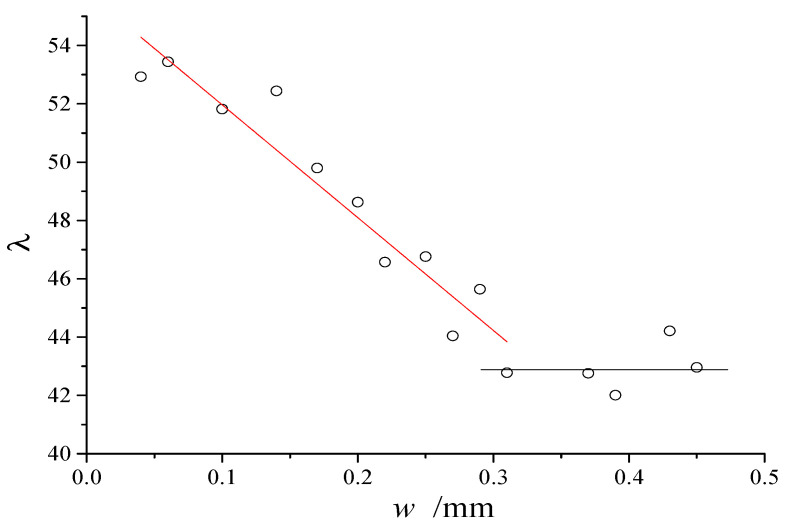
The relationship between *λ* and crack width by statistics.

Further, we calculate the λ once every 0.02 mm interval and count the relationship between λ and crack width, as shown in Figure 9. Interestingly, λ was decreasing linearly with the increase of crack width from 0.06 to 0.3 mm and was almost constant when the crack width was approximately 0.3 mm or more, shown as Equation (12).

(12) $$\left\{ \begin{array}{ll} \lambda =-39w+56 & 0.06\leq w<0.3\\ \lambda =43 & w\geq 0.3 \end{array}\right.$$

Since λ is a function of *h*, Equation (12) essentially shows that *h* and *w* are related. After analyzing the reasons for this, we believe it can be related to the constraint of the bottom of the concrete slab. In the crack developing process, the bottom of the concrete slab is always constrained, and this constraint will limit the development of the crack. The greater the crack depth, the greater the constraint applied, and the more difficult crack is to develop.

## 5. Results and Discussion

### 5.1. Theoretical Calculation of Equivalent Permeability of Shrinking-Cracked Concrete

For shrinking-cracked concrete cylinder specimen, the correlation between depth and width established in Section 4 is considered, which can be substituted in Equation (10), resulting in:

(13) $$\begin{array}{l} \frac{D_{eq}}{D_{0}}=\frac{\frac{4}{\pi }\frac{w}{b}\frac{D_{cr}}{D_{0}}+1}{\frac{4}{\pi }\frac{w}{b}\frac{D_{cr}}{D_{0}}\left(1-\frac{\lambda w}{H}\right)+1}\;(a)\;\mathrm{OR}\\ \left\{ \begin{array}{lll} \frac{D_{eq}}{D_{0}}=\frac{\frac{4}{\pi }\frac{w}{b}\frac{D_{cr}}{D_{0}}+1}{\frac{4}{\pi }\frac{w}{b}\frac{D_{cr}}{D_{0}}\left(1-\frac{56w-39w^{2}}{H}\right)+1} & 0.06\leq w<0.3 & \left(b\right)\\ \frac{D_{eq}}{D_{0}}=\frac{\frac{4}{\pi }\frac{w}{b}\frac{D_{cr}}{D_{0}}+1}{\frac{4}{\pi }\frac{w}{b}\frac{D_{cr}}{D_{0}}\left(1-\frac{43w}{H}\right)+1} & w\geq 0.3 & \left(c\right) \end{array}\right. \end{array}$$

**Figure 10:**
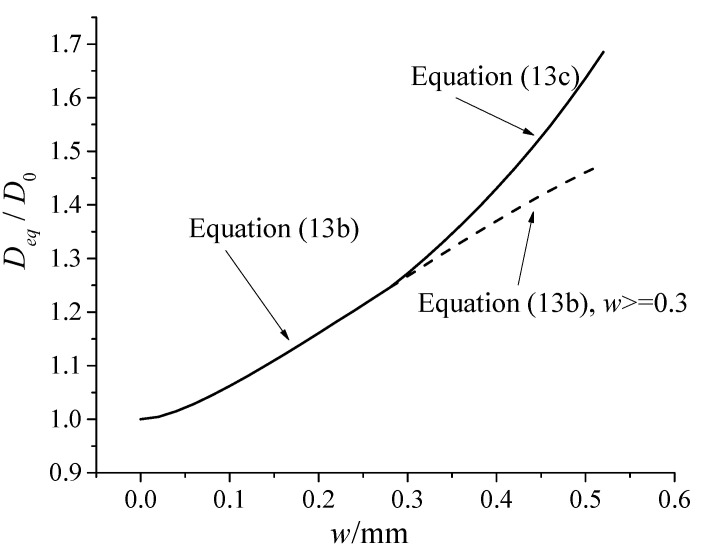
The variation of *D_eq_/D*_0_ versus the crack width *w* obtained from Equation (13).

The variation of equivalent diffusivity of shrinking-cracked concrete (*D_eq_/D*_0_) is shown by the solid line in Figure 10. As can be seen, it shows a totally different rule from Figure 3. When *w* increases, *D_eq_/D*_0_ increases more rapidly. The dotted line in Figure 10 indicates the relationship represented by Equation (13) when w ≥ 0.3. It can be seen that the change of λ of the shrinkage crack significantly affects the development of the equivalent permeability coefficient of concrete. 

### 5.2. Effect of Shrinkage Cracks on Diffusion Coefficient

**Figure 11:**
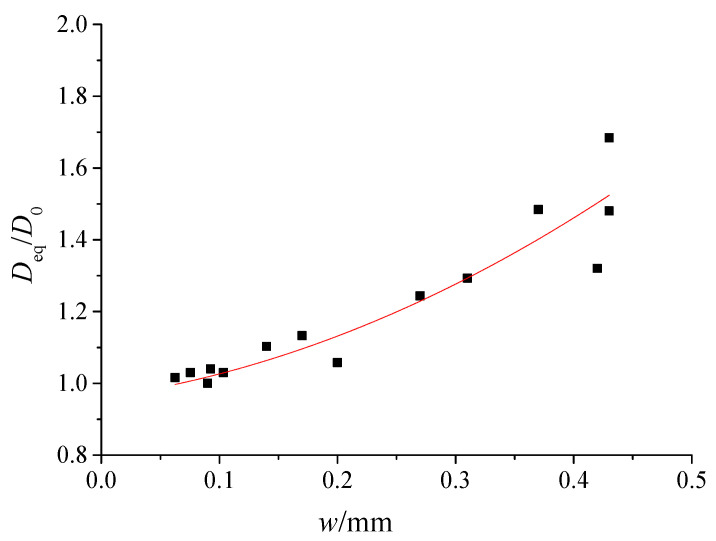
The variation of the ratio *D_eq_/D*_0_ versus the crack width obtained from tests.

The test results of chloride diffusion through shrinking-cracked concrete are shown in Table 3 and Figure 11, where the diffusion coefficients are normalized against that of the sound concrete (*D*_0_ = 1.49 × 10^−12^ m^2^/s). The results indicate that the coefficient *D_eq_/D*_0_ of cracked specimens is on the rise with the increasing crack width. In addition, their relationship is nonlinear, which is in accordance with the law of Equation (13) or Figure 10.

**Figure 12:**
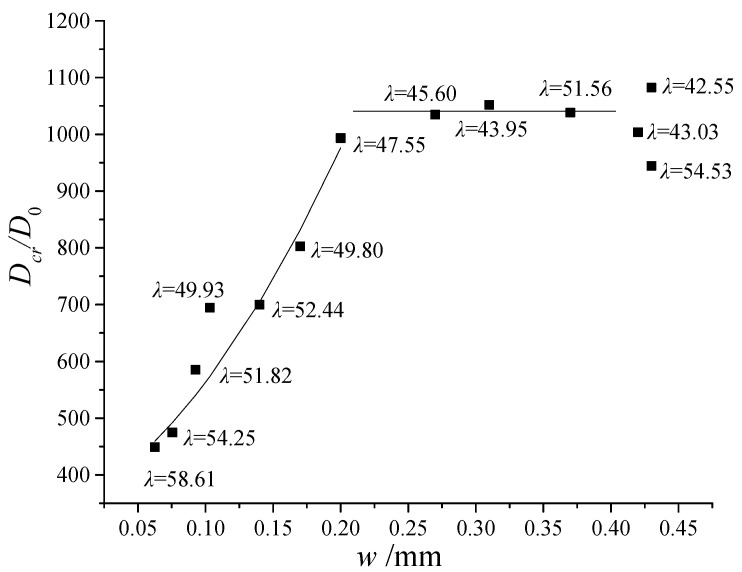
Effect of *w* and λ on diffusion coefficient through the crack.

When Equation (13) is substituted with the data in Table 3, the diffusion coefficient through crack *D_cr_* may be obtained. Figure 12 shows the relationship between *w*, λ and *D_cr_/D*_0_. *D_cr_/D*_0_ is increasing approximately squared with the increasing of crack width from 0.06 to 0.2 mm (meanwhile, λ was decreasing from 58.61 to 47.55, Equation (12)) and was almost constant when the crack width was approximately 0.2 mm or more.

It should be noted that λ also significantly affects the permeability of the crack. As a special exception to the statistical law, when the crack width is essentially constant (0.42–0.43 mm) and *λ* increases from 42.55 to 54.53, the crack permeability coefficient is greatly reduced by 13%. Further, it is easy to know that the diffusivity at the crack is about 0.36–0.87 times of that in free solution (2.032 × 10^−9^ m^2^/s at 25 °C), which is larger than those reported by Djerbi et al. [8]. This difference may be probably attributed to the tortuous characteristics of shrinkage cracks. Unlike the traversing cracks, shrinkage cracks are less tortuous due to the relatively small depth.

### 5.3. The “Threshold Crack Width” and Allowable Crack Width

The “threshold crack width” for diffusion is 0.08 mm found by Jang et al. [12] and Djerbi et al. [8], and 0.055 mm by Gagné et al. [17], and 0.4 mm by Zhang [28]. In the present study it is 0.2 mm. The conclusions between different studies vary greatly. There are two reasons for this problem. One is that those cracks are produced in different ways. Compared with the shrinkage cracks, the splitting cracks [8,12,17] were healed after unloading, and the width measured would be relatively small. The second is that the splitting cracks are all traversing while the uniaxial-compressing cracks and shrinkage cracks are not. According to the test results of this paper, the increasing of crack depth will significantly increase the permeability of cracked concrete, so the threshold width of splitting crack will be smaller than the uniaxial-compressing cracks and shrinkage cracks. 

Considering that, in actual engineering, concrete cracks may be generated by shrinkage or load, and cracks generally do not run through the protective layer, the limitation on crack width should be discussed separately. Assume that the thickness of the protective layer of concrete structure is 40 mm. For the shrinkage crack, based on the conclusions of the present study, when the crack width is 0.2 mm, λ*=* 48, *D_cr_*/*D*_0_ = 1020, and the equivalent permeability coefficient can be calculated by Equation (9), of which the change rate does not exceed 20%. For the load crack, refer to Zhang et al [29], the crack depth is affected by the height of the concrete beam and the reinforcement ratio. When the crack width is 0.2 mm, the crack depth is one third of the beam height (equivalent to λ*=* 67), and *D_cr_*/*D*_0_ = 700 found by Djerbi et al. [8], the change rate of equivalent permeability coefficient calculated by Equation (9) does not exceed 25%. Based on the above discussion, we believe that the limit of crack width allowable stipulated as 0.2 mm for concrete members in Chinese National Standards GB 50010 or CEB-FIP Model Code 90 is reasonable.

### 5.4. The Applicability of λ

Because of the difficulty in crack shape control, we cannot get a series of shrinkage cracks with different widths that we wanted. In order to obtain as many width shrinkage cracks as possible, only one mix proportion is designed for four concrete slabs in this paper. To assess whether the conclusion about λ obtained by us is applicable to other mixes of concrete, we use the experimental data of Reference [10] for an appropriate discussion. In Reference [10], mortar and cement paste were designed with the mix proportion of 0.5, which is different from this paper. According to its measured depth and width of the shrinkage cracks, we can calculate the range of λ is 33.9–89.8 and the average is 47.13, which is similar to the conclusion of this paper. Therefore, we believe that the influence of material on λ of shrinkage crack is not significant. Material change affects the absolute value of width and depth at the same time, while their relative changes might be constant. Moreover, λ is dimensionless, which could eliminate the effects of material changes. This belief might need further verification with additional experimental investigation.

## 6. Conclusions

The main conclusions of the present study are:

1. A series-parallel composite model was derived to evaluate the diffusivity of concrete with non-traversing cracks, and it shows that the equivalent diffusivity of cracked concrete is a function of crack depth and width for a crack, and it was relatively effected by the crack depth more than the crack width.
2. A drying-shrinking method on external restrained slab is proposed to generate shrinkage cracks, and the statistical analysis shows that the method can well simulate the generation of shrinkage cracks in concrete engineering.
3. The correlation between depth and width is obtained, which can be used easily to get the depth information (that is difficult to measure) by simply measuring the crack width, and is important for the prediction of chloride diffusion in shrinking-cracked concrete. The depth to width scaling of shrinkage crack increases with the crack width and was almost constant when the crack width was approximately 0.3 mm or more. Its average value is 44.90, and the crack tip angle is about 1–2 degrees.
4. The present study shows that the variation of equivalent diffusivity of shrinking cracked concrete presents a nonlinear rising growth trend with crack width, of which the correctness is verified by present test data.
5. The ratio *λ* significantly affects the diffusion coefficient through concrete cracks. As the ratio decreases with crack width, the diffusion coefficient increases. While the ratio increases and crack width is essentially constant, the permeability coefficient decreases. Therefore, attention must be paid to the width and *λ* characteristics of cracks for the permeability analysis of cracked concrete.
6. The calculation of chloride diffusivity in concrete is performed for shrinking crack and loading crack, respectively, which shows that a crack width of 0.2 mm has little effect on the permeability of concrete, and it is not necessary to impose a stricter limit on the concrete crack width.

## Figures and Tables

**Table 1:** Mix proportions of the concrete.

Specimen	Quantity (kg/m^3^)				*w*/*c*	Compressive Strength/MPa
	Cement	Water	Sand	Coarse Aggregate		
C40	402	185	599	1198	0.46	44.3

**Table 2:** The parameters for statistical analysis of λ.

Parameter	Value
Number of Data Sets	64
Maximum value	89.80
Minimum value	18.84
Average	44.90
Standard deviation	14.08
Histogram bars	13
Histogram group distance	6.00

**Table 3:** Measured diffusion coefficients for various test variables.

Number	Crack Width	*λ*	*D_eq_/D*_0_	*D_cr_/D*_0_
1	0.06	58.61	1.02	448.75
2	0.08	54.25	1.03	474.50
3	0.09	50.01	1.0002	-
4	0.09	51.82	1.04	585.37
5	0.10	49.93	1.03	694.69
6	0.11	49.90	0.95	-
7	0.14	52.44	1.10	699.72
8	0.17	49.80	1.13	802.90
9	0.20	47.55	1.06	993.36
10	0.27	45.60	1.24	1034.80
11	0.31	43.95	1.29	1052.19
12	0.37	51.56	1.48	1038.44
13	0.42	43.03	1.32	1003.89
14	0.43	42.55	1.48	1082.40
15	0.43	54.53	1.68	944.36
